# A Classification of Basic Helix-Loop-Helix Transcription Factors of Soybean

**DOI:** 10.1155/2015/603182

**Published:** 2015-02-11

**Authors:** Karen A. Hudson, Matthew E. Hudson

**Affiliations:** ^1^USDA-ARS Crop Production and Pest Control Research Unit, 915 West State Street, West Lafayette, IN 47907, USA; ^2^Department of Crop Sciences, University of Illinois, 1101 W. Peabody Drive, Urbana, IL 61801, USA

## Abstract

The complete genome sequence of soybean allows an unprecedented opportunity for the discovery of the genes controlling important traits. In particular, the potential functions of regulatory genes are a priority for analysis. The basic helix-loop-helix (bHLH) family of transcription factors is known to be involved in controlling a wide range of systems critical for crop adaptation and quality, including photosynthesis, light signalling, pigment biosynthesis, and seed pod development. Using a hidden Markov model search algorithm, 319 genes with basic helix-loop-helix transcription factor domains were identified within the soybean genome sequence. These were classified with respect to their predicted DNA binding potential, intron/exon structure, and the phylogeny of the bHLH domain. Evidence is presented that the vast majority (281) of these 319 soybean bHLH genes are expressed at the mRNA level. Of these soybean bHLH genes, 67% were found to exist in two or more homeologous copies. This dataset provides a framework for future studies on bHLH gene function in soybean. The challenge for future research remains to define functions for the bHLH factors encoded in the soybean genome, which may allow greater flexibility for genetic selection of growth and environmental adaptation in this widely grown crop.

## 1. Introduction

Basic helix-loop-helix (bHLH) transcription factors belong to a large family of genes present in the shared ancestor of plants, animals, and fungi, and this family has undergone an expansion in the land plant lineage [1]. Often referred to as helix-loop-helix (HLH) proteins, a loosely defined basic domain is involved in DNA binding [2] and present in the great majority of characterized proteins in this family [1]; thus the term bHLH factors is used henceforth. bHLH transcription factors have been implicated in numerous biological processes in plants including responses to light, cold, and hormones, epidermal cell fate determination, developmental patterning in roots and flowers, and anthocyanin biosynthesis [3–14]. In many cases, the bHLH family is critically important for correct developmental and environmental responses, as demonstrated by a large number of mutants in* Arabidopsis* with severe phenotypes as a result of a lesion in a bHLH-encoding gene. Development and dehiscence of the seed and seed pod (silique) [13, 15, 16] and responses to light quality and photoperiod [9, 17–21] are particularly known to be under the control of bHLH factors, and these phenomena are important to soybean agronomic performance. Characterization of the bHLH-encoding gene family can therefore be a useful step in the detailed functional characterization of the soybean genome.

The bHLH transcription factors have been extensively characterized at the sequence and structural level. In animals, the best-known and most thoroughly characterized bHLHs are well-known regulators and proto-oncogenes such as c-Myc, Max, and E47, where in many cases structural data on the proteins and their interaction with DNA molecules is available [2]. Many animal bHLHs show a binding preference for the so-called “E-box” motif (CANNTG) and the residues within the protein that are required for sequence specific recognition are well defined (reviewed in [2, 22, 23]). A number of plant bHLH proteins have been demonstrated to show a particular preference for binding the G-box (CACGTG) sequence (a subset of E-box) [3, 19, 24–27]. Homo- and heterodimer formation are also ubiquitous and required for DNA binding within the bHLH family, a property that increases the combinatorial possibilities for regulation of transcription. The *α*-helices in the bHLH domain and the other motifs outside the conserved bHLH domain are required for protein-protein interactions and function [28–32].

Classification of bHLH proteins is usually based on sequence homology within the conserved bHLH domain. In recent years, a number of proteins have been identified genetically that represent novel and atypical bHLH proteins that were not previously classified by homology-based approaches, in some cases because a basic domain was lacking [31, 33–36]. An intriguing feature of the bHLH family is that the proteins can often be very divergent outside of the highly conserved bHLH domain and contain a range of other motifs, not all of which have known functions [1, 37]. For this reason, a sequence-homology-based search approach using the entire gene sequence (such as BLAST of the canonical bHLHs from* Arabidopsis*) may not be the most appropriate tool to identify all the bHLH-encoding genes in a genome such as soybean. An alternative approach is to use data from the conserved HLH motif across the kingdoms of life to develop a hidden Markov model (HMM) that allows the detection of the bHLH domain across highly divergent sequences without the need for extensive sequence identity [38]. This type of approach has been successfully deployed for identifying conserved motifs in distantly related proteins and allows more sensitive and accurate discovery of a specific motif like the bHLH domain. The PFAM project (http://pfam.sanger.ac.uk/) uses a curated alignment approach to provide constantly updated HMMs for most characterized protein families, including the HLHs. HMMs for the subsidiary motifs of the different bHLH families can be generated using protein alignments.

Several recent genome-wide studies have examined and classified the bHLH protein family in* Arabidopsis thaliana*, rice, poplar, and other plants with whole-genome sequences, but these studies have not examined the conservation and diversification of this family in the soybean [1, 33, 37, 39–42]. The bHLH transcription factor family is the second largest family of transcription factors in plants (behind the myb family), and the complete whole-genome sequence of soybean revealed a number of genes predicted to encode bHLH proteins [43–45]. A study of this family of genes in soybean and classification of the bHLHs into subfamilies orthologous with those present in other species is useful in order to provide a list of candidate genes that are likely to be upstream regulators of a number of processes. Such lists of candidate genes enable association mapping approaches to proceed with knowledge of potential functions for regulatory genes likely involved in conferring seed and agronomic traits [46]. They are also very helpful as a means to rapidly identify candidate genes within positional genomic intervals defined by conventional genetic fine mapping in mutant studies or as quantitative trait loci (QTL) in recombinant inbred populations of soybean.

## 2. Materials and Methods

### 2.1. Identification of bHLH Sequences Using Hidden Markov Models

Soybean,* Arabidopsis*, and rice gene models were originally identified from annotation versions contained in the Phytozome v7.0 package. The most recent version of the soybean assembly (Assembly v.2.0 and gene models for Phytozome v.10) was compared with our data; however since stringent new rules for inclusion of genes in the newer assembly and annotation resulted in the loss of 62 conserved bHLH genes including several with confirmed expression data, the legacy annotation and assembly were retained for this analysis (http://www.phytozome.net/) [44, 47, 48]. To identify bHLH transcription factors in the soybean genome, a hidden Markov model search (hmmsearch v. 3.0; http://hmmer.janelia.org/) was applied to the predicted open reading frames (ORFs) of soybean (genome and assembly version Gmax109; [44]) using the PFAM HLH hidden Markov model (PF00010; http://pfam.sanger.ac.uk). No *e*-value cutoff was initially applied in order to maximize detection of poorly annotated and orphan bHLH sequences. A cutoff was applied during manual curation (see next section). A total of 329 hits to unique predicted proteins containing putative HLH domains were initially identified. These included 31 ORFs not annotated as bHLHs by the soybean genome annotation and a small group of HLH proteins that lack the basic domain (see Section 3). Data on expression of soybean bHLH genes was obtained from http://www.soybase.org/ and is described in [49].

### 2.2. Curation of bHLH Sequences

Sequences were manually curated to identify mispredictions of splice sites, which often led to omission of part of the bHLH domain in the genome annotation, reducing the hidden Markov model score. Sequences that included in-frame stop codons in the Williams-82 genomic sequence or were missing part of the HLH domain were removed, and these soybean gene models are listed in Additional File 2 (see Additional File 2 in Supplementary Material available online at http://dx.doi.org/10.1155/2015/603182). We then examined the revised list for sequences that were atypical or were unlikely to be true bHLHs. Several studies have used a stringent cutoff restricting the number of mismatches to the canonical bHLH motif found in mammals [22, 39, 40, 50]. Other studies have used a less stringent cutoff to identify additional, atypical bHLHs in characterized genomes [33]. Empirically, it was observed in the soybean bHLH domains that such a cutoff of 6 mismatches to the core 11 residues or 11 mismatches to the larger 19 defined residues [51] would eliminate the soybean homologs of functionally characterized, bHLH-related proteins, and this also corresponded to the maximum number of mismatches observed in the soybean bHLH domains. Therefore, no cutoffs were used, other than those previously described (i.e., the HMM search had to hit the sequence (at any *e*-value) and no sequences with incomplete bHLH domains or that contained stop codons were included).

### 2.3. Multiple Sequence Alignment

Alignments were performed using MAFFT v.6.811b [52] with the following parameters: alignments were visualized and edited using Geneious Pro v.5.4.6 for the Macintosh (Biomatters Ltd., Auckland, New Zealand).

### 2.4. Phylogenetic Analysis

The construction of the bootstrapped maximum likelihood phylogenetic tree was performed using the HPC-MPI version of RAxML version 7.3.0 [53] with Geneious used to manage, curate, and reformat the MAFFT alignment files. The program was run in an MPI environment on 96 processors. The PROTCAT option was used to select the appropriate model, and appropriate gamma model parameters were automatically selected by the software as was a maximum likelihood estimate of 25 per-site rate categories. The Dayhoff substitution matrix was used. 1052 bootstrap trees were generated, and the highest-scoring tree was visualized using the TreeExplorer utility of MEGA 5.0 and used together with bootstrap confidence values (as a percentage of trees) to create the figures. All trees were unrooted.

### 2.5. Secondary Motif Detection

Assignment of subsidiary motifs in the bHLH or HLH proteins was performed using the alignment of motifs provided in Supplemental Material by Pires and Dolan [1]. Hidden Markov models were generated from script-reformatted versions of these alignments using hmmbuild 2.3.2 (the 3.0 version of hmmbuild lacks import filters for alignments in formats other than Stockholm or SELEX [54]). The presence of motifs was detected by running the full-length predicted proteins against each model using hmmsearch as described above, using a bash shell script for automation. For the APB domain, the sequences in the alignment described by Khanna et al. [55] were realigned (using MAFFT* -*-auto* -*-reorder* -*-clustalout) and models generated and used for searching as described for the other motifs.

### 2.6. MEME Search and New Motif Detection

Using MEME (http://meme.sdsc.edu) we searched for up to 10 new sites between 2 and 300 residues wide. Using “discriminative motif discovery” a file was supplied containing the bHLH motif plus the sequences used to create the hidden Markov models from the known bHLH secondary motifs. A single strongly significant motif, Motif 40, was detected. As above, the alignment supplied by MEME for the new motif was used to create a hidden Markov model, and this model was used to search the soybean bHLHs to determine which of them contained the motif. As before no *e*-value cutoff was applied, however the Family X sequences all showed *e* < 10^−7^ while the two Family IX sequences GmbHLH262 and 261 showed *e*-values of 0.0025 and 0.003, respectively.

## 3. Results

### 3.1. The bHLH Domain Is Highly Conserved in Soybean bHLH Transcription Factors

**Figure 1:**
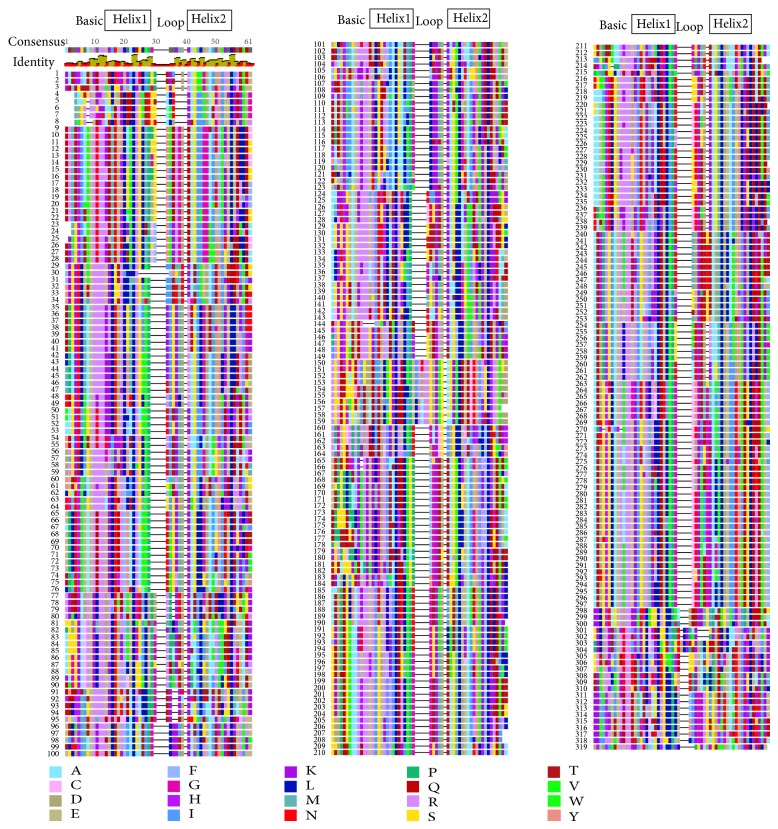
Alignment of the bHLH domain from 319 soybean proteins. The bHLH region was identified using a HLH hidden Markov model and trimmed or extended from soybean predicted proteins to fit the canonical region aligned in this figure and then aligned using a fast Fourier transform algorithm. A color key is used for the 20 amino acid residues. Extent of conservation and a consensus sequence are shown at top left. The basic and helix-loop-helix regions can be seen via the annotations and the color key. The consensus graph indicates 100% conserved residues as green (there are none in this alignment), 30% or more conserved residues as yellow, and less than 30% conserved residues as red. A full text version is available in Additional File 3.

In total, 319 gene models were identified as encoding bHLH transcription factors in soybean (see Section 2). Each soybean bHLH sequence in the alignment was assigned a number, as is the convention in other species [41]. A table showing the correspondence of the GmbHLH numbers to the soybean gene models from Glyma version 1.1 as well as version 2.0 and other bHLH classification information in tabular form can be found in Additional File 1. The bHLH domain consists of an N-terminal basic region of approximately 13 amino acids, followed by two alpha helices (14-15 residues in length) separated by a loop that ranges from 5 to 14 amino acids (Figure 1, Additional File 3). (Position numbers in Figure 1 and Additional File 3 follow the convention of [22], with the exception of the numbering of the second HLH domain which is numbered consistent with our soybean alignment.) This pattern is conserved in multiple plant species as well as soybean [50, 51]. Within the two *α*-helices, several hydrophobic residues are thought to be required to stabilize the secondary structure of the protein, and these residues are highly conserved in both plant and animal sequences [22, 23]. At position 23, over 99% of soybean bHLHs have a characteristic L residue. At position 55, 96.8% of soybean bHLHs contain L residue. Positions 45 and 52 are occupied by either I, L, or V, in 98.4% and 94% of soybean bHLH proteins, respectively (Figure 1). Additional File 3 contains a full text multiple sequence alignment for all of the soybean bHLH domains.

The conservation of key residues within the basic region of the bHLH domain can predict both the potential to bind DNA and the preferred recognition sequence. The E residue at position 9 within the basic domain has been shown in protein crystal structures to make contact with the major groove of the DNA (reviewed in [23]). This position is conserved in 245 of 319 of the bHLHs identified in soybean (77%) and has previously been shown to be present in 74% of bHLH sequences from other plants [1]. It has been shown that E at position 9 and R at position 12 are required for the recognition of an E-box sequence [56]—244 of the soybean bHLHs have this configuration and can be classed as E-box binding. Another pattern has been described that includes H or K at position 5, E at position 9, and R at position 13 (H_5_-E_9_-R_13_); in animals these bHLHs recognize the E-box subset CACGTG [22]. This sequence is a commonly occurring promoter motif in plant genomes, where it is referred to as the G-box, and a number of plant bHLH proteins have demonstrated G-box binding activity [3, 24, 26, 27]. 186 (58%) of soybean bHLH proteins have a conserved H/K_5_-E_9_-R_13_ motif and thus could be candidates for binding the G-box sequence. The conservation of these residues that are potentially involved in protein-DNA interaction within soybean is an indication that the DNA-binding function and also the recognition sequence may be conserved.

Of the soybean bHLH proteins that lack the E_9_ residue (74 proteins) only eight have five or more basic residues within the basic region. The number of basic residues has been previously used as a criterion to classify HLH proteins as having the potential to bind DNA [40]. Since the E_9_ residue is thought to be required for E-box binding, it is possible that these proteins bind DNA at a non-E-box DNA sequence, although this has not yet been demonstrated for plant bHLHs [40]. The remaining 66 HLH domains, which contain 4 or fewer basic residues within the basic region, are classified as HLH proteins and are unlikely to bind DNA directly. Many proteins identified through mutant studies in recent years in plants as “atypical” or nonbasic bHLHs have few or no basic residues in this region [7, 31, 34, 35, 57]. They do not bind DNA but in some cases act in concert with the more typical bHLHs to regulate gene expression by negative interference, and similar non-DNA binding HLHs exist in animal systems [22, 31, 58, 59]. Since these helix-loop-helixes are related to the bHLHs and are involved in many of the same pathways, they are included in our characterization of soybean bHLHs if they are recognized by the HLH HMM.

**Figure 2:**
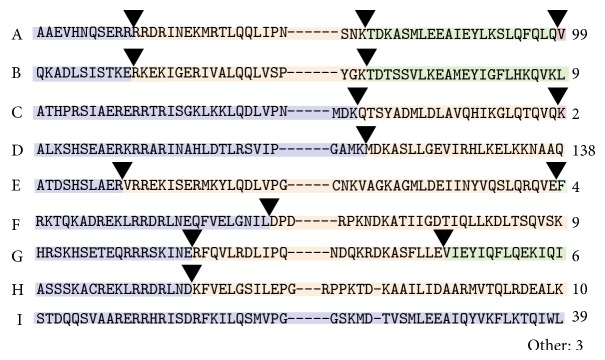
Conservation of intron position within the bHLH domain of soybean bHLH genes. Representative bHLH sequences are shown with the positions of three typical bHLH introns (pattern A) marked and the number of genes (99) that fit this pattern. Other bHLH genes have a subset of these three introns (patterns B–E). An alternative pattern F contains one intron at a different location. The genes with pattern G have two other conserved introns, and pattern H has only the first of these introns. 39 of the soybean bHLH genes have no introns within the bHLH domain. Three other bHLH genes exhibit a different intron-exon pattern. The specific domains shown are as follows (A–I): GmbHLH234, GmbHLH183, GmbHLH253, GmbHLH136, GmbHLH289, GmbHLH77, GmbHLH145, GmbHLH84, and GmbHLH98. Intron patterns for all soybean bHLHs are located in Additional File 1.

Most plant bHLHs have a conserved intron structure with three introns within the bHLH domain [33, 39, 40]. The intron/exon patterns were examined for soybean bHLH-encoding genes, and it was determined that 31% of soybean bHLHs contain the pattern of three conserved introns (pattern A, Figure 2). 43% of the soybean bHLHs contain only one of these introns (pattern D), and 4% of soybean bHLHs have another subset of these conserved introns (patterns B, C, and E). Only 8.7% of soybean bHLHs exhibit a different splicing pattern, which fall into other distinct classes (Figure 2, patterns F, G, H, or other). 12% of soybean bHLHs have no introns within the bHLH domain. The proportions of distinct intron patterns in soybean genes are consistent with the intron structure distribution in the* Arabidopsis* and rice bHLH families [33, 39, 40]. As previously observed for the bHLH superfamily, intron distribution tends to be conserved within bHLH subfamilies and lends additional credence to these class distinctions. The intron pattern for each soybean bHLH is listed in Additional File 1.

### 3.2. Phylogenetic Relationships of Soybean bHLH Domains

**Figure 3:**
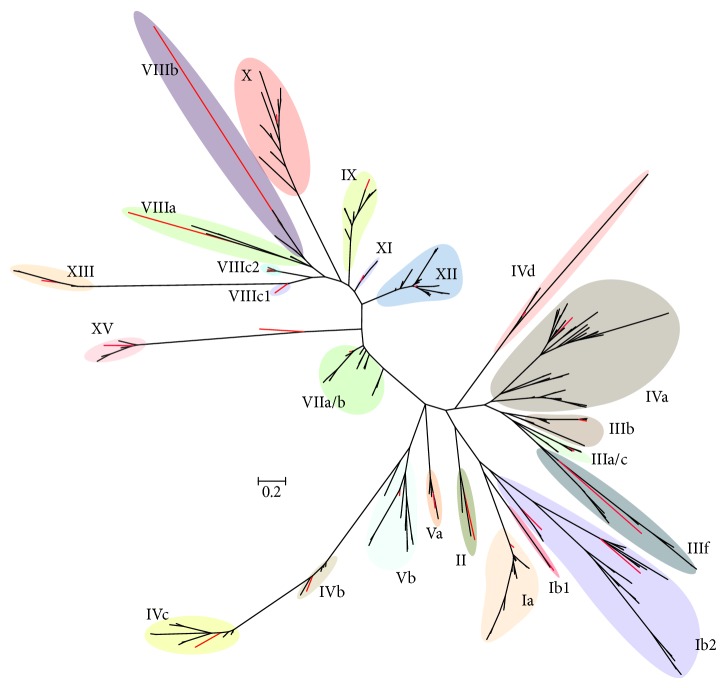
Maximum likelihood phylogenetic tree of soybean bHLH gene families. A radiation diagram is used to represent an unrooted maximum likelihood tree (highest scoring of 1052 bootstraps) with branch lengths proportional to estimated sequence distance. Colored balloons represent named bHLH families identified by sequence similarity to the families in* Arabidopsis*, and red branches represent* Arabidopsis* bHLHs included to identify the families. Scale bar represents 0.2 expected amino acid residue substitutions per site. A full version of this tree with individual bHLH sequence names and bootstrap support values can be found in Additional File 4.

The bHLH superfamily in plants is composed of between 14 and 32 subfamilies based on phylogenetic analysis of the bHLH region [1, 33, 37, 39, 40]. Supporting these classifications, it has been found that both the intron patterns, other domains of sequence homology outside the bHLH region, and DNA binding potential are often conserved within these subfamilies. A phylogenetic reconstruction of the soybean bHLHs shown in Figure 1, together with at least one* Arabidopsis* bHLH sequence representing each of the major subfamilies, was generated based on the alignment of the bHLH domain (Figure 1 and Additional File 3). The alignment used to generate the phylogenetic tree, which contains representative* Arabidopsis* sequences and excludes all but one of any identical soybean sequences, is supplied as Additional File 7. A bootstrapped maximum likelihood tree (1,052 bootstraps) was constructed from this alignment using RAxML [53]. The best scoring tree is displayed in Figure 3 using a summary radiation diagram to show branch lengths and provide an overview of the similarities within 24 bHLH subfamilies found in soybean. The full phylogeny including bootstrap support values (expressed as percentages) is presented in Additional File 4. A number of intriguing aspects of the soybean bHLH proteins are apparent from this tree. Firstly, soybean appears not to contain any representatives of Family XIV. Family XIV has one functionally characterized member in* Arabidopsis*, SAC51/AtbHLH142, which is involved in spermidine synthase-mediated stem elongation [60]. Secondly, Family VIIa/b has been repeatedly shown to be involved in light signalling [9, 17–21]. Interestingly, PIF3 [20] (AtbHLH008), which interacts directly with phytochromes and mediates light-responsive gene expression, has a number of highly conserved homologs in soybean Family VIIa/b. The short branch lengths within this family may indicate higher levels of sequence or structural constraint. However, HFR1 [29] (AtbHLH026), also involved in light signalling and normally placed in Family VIIa/b, has only very distant similarity to any soybean protein and, in our analysis, appears as an outlier of Family XV (Figure 3, Additional File 4). HFR1 lacks a clear basic domain and may not be able to bind DNA [29]. We did not observe any atypical bHLH proteins or families in soybean that clearly do not fit into one of the characterized* Arabidopsis* families; however several ambiguous sequences were observed in the phylogeny (Figure 3, Additional File 4). We were able to assign families to all these sequences using branch length data, additional motif information, and BLAST searches; however not all families formed monophyletic groups (see Additional File 4).

In addition to the bHLH domain analysis, any of the bHLH subfamilies can also be distinguished by the presence of one or more characteristic motifs outside the bHLH domain [1, 33, 37, 55]. To identify these motifs in the predicted full-length sequences of the soybean bHLHs, HMMs were created from alignments of the motifs from across the plant kingdom that were previously published [1]. The soybean bHLHs and subfamilies that possess these defined motifs are highlighted in Additional File 1. All of the previously described motifs were detected with the exception of Motif 28, which is associated with Family XIV, which was also found to be absent from soybean. The HMM created from the alignment of the active phytochrome binding (APB) domain described in [55] matched precisely the same protein motifs as those identified using the HMM for Motif 14 [1]. A search was conducted using MEME (http://meme.sdsc.edu) for new motifs, by excluding the known secondary motifs plus the bHLH domain. A single motif was detected with the consensus sequence GLCLVPVScTqqVgseNGADYWAPayggg (Additional File 8). This sequence is strongly conserved in all the GmbHLH sequences 165–184 (Family X). In the Supplemental Material this is named Motif 40, although it follows closely the distribution of Motif 20; the NGADYWAP portion of this motif is similar to Motif 20 and it likely represents an expanded version of this motif. Much weaker similarity to Motif 40 is also observed in GmbHLH261 and GmbHLH262 of Family XI. This motif is also conserved in several* Arabidopsis* bHLHs in Family X.

### 3.3. Soybean bHLH Proteins Have Close Homologs in Other Plants

To compare the soybean bHLHs to* Arabidopsis* and* O. sativa* homologs, BLAST searches of the predicted* Arabidopsis* and rice proteomes were conducted with the predicted full-length coding sequences of the soybean bHLHs [47]. A total of 141 soybean bHLHs hit* Arabidopsis* proteins with >50% amino acid sequence identity, and 288 had hits with an *e*-value of less than 10^−25^ (Additional File 5). A total of 151 soybean bHLHs shared >50% sequence identity with rice, 255 with an *e*-value of less than 10^−25^. In order to increase confidence that true orthologs were detected, a reciprocal BLAST search of the soybean genome was performed using full-length* Arabidopsis* and rice bHLH sequences, and the orthologs are presented in Additional File 5. Of the* Arabidopsis* bHLHs identified as matching soybean bHLHs, all but one of the* Arabidopsis* sequences are known from previous classification of bHLH proteins in* Arabidopsis* [33, 37, 40]. The one* Arabidopsis* protein not previously classified as a bHLH is At2g40435, a protein with homology to 5 soybean HLH proteins. The soybean proteins lack a complete basic domain, show a reasonably conserved HLH motif but have some divergence from previously described consensus sequences, and match the HLH HMM with relatively low scores. At2g40435 has strong similarity to these soybean proteins in this predicted HLH region, but HMMER does not find a match to the HLH HMM at all. Five rice proteins (corresponding to 16 soybean genes, all of which were identified as similar to known* Arabidopsis* bHLHs) were also not in previous classifications of rice bHLH proteins (Additional File 5) [33, 39]. All but one of these proteins have a HLH domain predicted by the HMM search as used here for soybean; the fifth has no HMM similarity to the HLH but does have similarity to the HLHMycN conserved domain.

### 3.4. bHLHs and Genome Duplication

Because of the recent duplication of the soybean genome [44], many soybean bHLH proteins have closely related homeologs (recently duplicated paralogs arising as a result of polyploidy) which may be as much as 98% identical at the DNA sequence level. To determine which bHLHs were recent homeologs, we combined the data on recently duplicated genomic regions of soybean and validated potential homeologs by sequence identity (tabulated data provided by Dr. Jessica Schuleter of the University of North Carolina, Charlotte) [44]. 213 of the soybean bHLHs exist in more than one homeologous copy. 135 are present as two copy loci, 21 are present in three copies, 52 are present in 4 copies, and 6 are present in 6 copies. Information on the homeologous groups of soybean bHLHs is included in the table in Additional File 1.

### 3.5. Expression of bHLHs in Soybean

**Figure 4:**
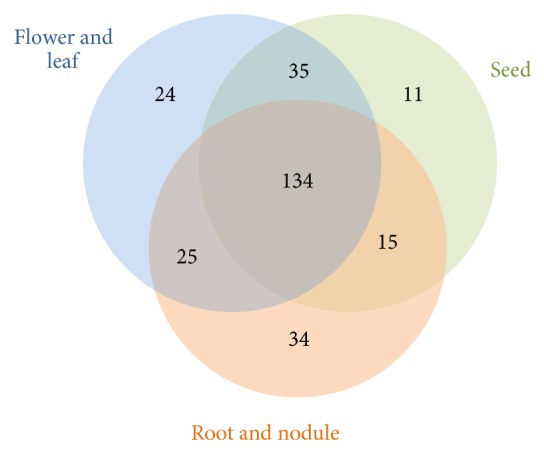
Summarized expression data for soybean bHLHs. 281 soybean bHLHs were found to be expressed. Root and nodule expression classes were combined as all, but 11 of the 150 nodule-expressed bHLHs were found to also be represented in the root class. Three of the total expressed bHLHs were detected only in pod wall tissues (see Additional File 1).

Using a survey of the publicly available transcriptome data for soybean [49], it was investigated what fraction of the soybean bHLHs were actively expressed at some stage of plant development. It was determined that 281 of 319 (88%) bHLHs showed some evidence of expression at the mRNA level (Figure 4). 47% of these are expressed across root and leaf, as well as developing seed tissues, while the remaining genes showed some evidence of tissue specific expression (one or more tissue types). Notably, this included 5 soybean bHLH mRNAs that are expressed preferentially in nodules and are mostly absent from aerial tissues (Additional File 6). The closest* Arabidopsis* orthologs of these preferentially nodule-expressed bHLHs do not have known biological functions and do not cluster in any particular subfamily. Fifteen bHLH transcription factors are enriched in expression during soybean seed development. Among these are homologs of* TARGET OF MONOPTEROS 5* and* TRANSPARENT TESTA 8*, both of which have known roles in* Arabidopsis* embryo development (Additional File 6) [61, 62].

## 4. Discussion

The whole-genome duplication events in soybean that occurred 59 million years ago and 13 million years ago have clearly had a substantial impact on the size of the bHLH family in soybean when compared to other plant species [1, 37, 39, 40, 44]. Most of the soybean bHLHs (213) were present in homeologous copies duplicated two or more times. Expression evidence was identified for 281 of the bHLH genes, and 122 bHLHs were expressed in leaf, seed, and root tissue. Fully 106 genes are present in only a single copy. These single-copy genes are likely the result of deletion of the homeolog after whole-genome duplication, perhaps because of negative selection due to deleterious effects from multigene dosage. Speculatively, these genes may be involved in critical developmental or other processes in which these deleterious effects are seriously disadvantageous, and thus selection for loss of the homeologous copy has already taken place. Several of the genes with strong evidence for expression and/or conservation were omitted from the current soybean genome annotation, suggesting that this version of the annotation omits a relatively large number of bHLH genes.

Based on sequence conservation within the basic domain, it can be predicted that the majority of soybean bHLHs (77%) have the potential to bind DNA, and 58% of all soybean bHLHs are likely to recognize the core G-box sequence, of which there are over 17,000 represented in the promoters of actively transcribed genes in the soybean genome (MEH, unpublished data). There is experimental evidence that different bHLH proteins have a preference for certain nucleotides flanking the core G-box sequence and that residues in the second *α*-helix may also be involved in DNA contact, increasing specificity of genomic sequence targets [24, 56, 63]. All but one of the bHLH subfamilies represented in other land plants are present in soybean, and the proportion of bHLHs in each subfamily was largely consistent between* Arabidopsis* and soybean, although soybean contains many more bHLH-encoding genes, implying a broadly similar set of functions for each bHLH family may be conserved. All but one of the conserved motifs identified in bHLH subfamilies were found in soybean bHLH genes. A small number of these motifs are known to be involved interactions with other proteins (such as the APB and leucine zipper motifs) [32, 55].

Using MEME a long, highly conserved motif was identified, which we term Motif 40. This sequence is strongly conserved in all Family X bHLHs in soybean. Much weaker similarity was found in two Family XI proteins. The sequence is also strongly conserved in* Arabidopsis* Family X proteins. The strongly conserved NGADYWAP portion of this motif is similar to Motif 20, with which it shares very similar distribution. Motif 40 also contains the GLCL sequence contained in Motif 22. We do not believe Motif 40 is related to Motif 22 because flanking conserved residues of Motif 22 are very different to those of Motif 40, and because the distribution of Motif 40 is strongly correlated with and predictive of membership in Family X. Motif 20 also correlates with Family X but is not found in all members with strong significance, perhaps due to greater discriminative power and sensitivity for the longer motif.

Recently a number of “atypical” bHLH genes have been described. The HMM method identified the bHLH domain in the closest soybean homologs of several of these genes including* KIDARI*,* ATBS1*, and* PRE1*, and these were used in our analysis. In the case of* LONESOME HIGHWAY*, a clear ortholog was present in soybean (Glyma12g31460, at a BLASTP *e*-value of 10^−134^) but this gene was not predicted to contain a bHLH domain using our HMM search. In the case of* PAR1* and* PAR2*, by contrast, no homolog was apparent within the soybean bHLH proteins identified here using the HMM. While* PAR1 *and* PAR2* have BLAST hits in soybean, they are not clearly orthologs. The BLASTP *e* values of the best hits are in the range 10^−15^ to 10^−16^, which represents strong similarity but is a lower level of confidence than for the orthologs of other genes such as* KIDARI*. This may indicate that the putative HLH domains of* LONESOME HIGHWAY, PAR1,* and* PAR2* are divergent to an extent where structural similarity to canonical bHLHs is limited, or it could indicate that the HMM we used is not sensitive to this type of HLH domain, since it did not include representatives of this type of atypical bHLH.

However, five soybean predicted HLH genes were identified, highly similar to an* Arabidopsis* gene (with greater than 50% amino acid sequence identity and BLAST *e*-value of less than 2*e* − 33) not within the set of classified bHLH proteins. Interestingly, while all five soybean proteins are predicted to contain a HLH domain using the HMM approach, the same method does not predict a bHLH domain in the highly similar At2g40435 protein. The amino acid similarity between these proteins is very strong in the predicted HLH region of the soybean proteins, and the HMM score for the HLH prediction in the soybean proteins is not strong. The putative HLH domain is located at the extreme N-terminus of the soybean proteins, which fall into bHLH subfamily III but are not predicted to bind DNA. The At2g40435 protein is thus a candidate for an atypical bHLH. The intriguingly high level of sequence conservation in these genes across* Arabidopsis* and soybean may point to an important, previously unknown biological function, possibly connected with the bHLH similarity.

## 5. Conclusions

We have identified a large number of candidate genes from a family with likely regulatory roles in many processes critical for soybean growth and genetic improvement. These results establish a foundation for future functional genomics studies to target bHLH-controlled processes for modification and improvement in soybean.

## Supplementary Material

As Supplementary Material we have included the following. Addititional File 1: an Excel spreadsheet file that includes names and expression data for all of the soybean bHLH-encoding gene models.
Additional File 2: A list of soybean gene models excluded from the analysis due to an incomplete bHLH domain or predicted in-frame stop codon.
Additional File 3: A full-text alignment of all 319 soybean bHLH domains in pdf format.
Additional File 4: a detailed phylogenetic tree of the soybean bHLH genes including full names and predicted family membership.
Additional File 5: An Excel spreadsheet showing the presumptive rice and Arabidopsis orthologs of the soybean bHLH genes.
Additional File 6: An Excel spreadsheet containing a list of bHLHs enriched for expression in seed or nodules.
Additional File 7: The alignment of 319 soybean bHLH genes in PHYLIP format for computer analysis.
Additional File 8: A LOGO illustration of the putative Motif 40.
