# Supplementary material for: A Classification of Basic Helix-Loop-Helix Transcription Factors of Soybean
Source: Int J Genomics. 2015 Feb 11;2015:603182. doi: 10.1155/2015/603182 (PMC4339708; doi:10.1155/2015/603182)

Additional File 3. Multiple sequence alignment for 319 soybean bHLH domains

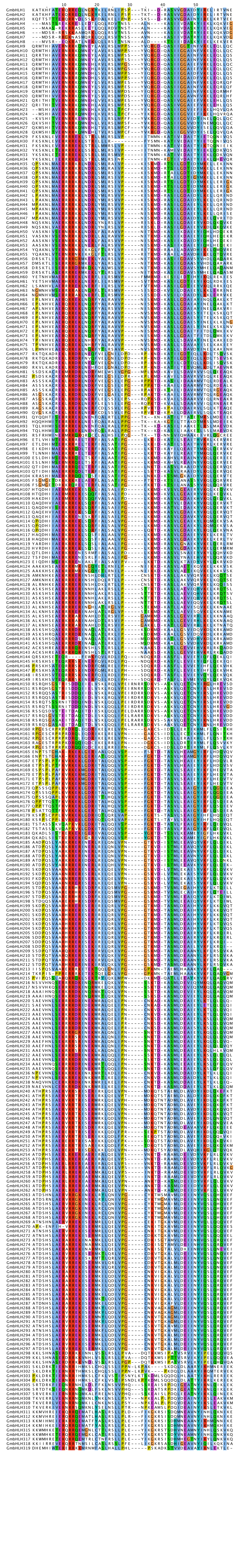

Supplement: Supplementary file 1 — As Supplementary Material we have included the following. Addititional File 1: an Excel spreadsheet file that includes names and expression data for all of the soybean bHLH-encoding gene models. Additional File 2: A list of soybean gene models excluded from the analysis due to an incomplete bHLH domain or predicted in-frame stop codon. Additional File 3: A full-text alignment of all 319 soybean bHLH domains in pdf format. Additional File 4: a detailed phylogenetic tree of the soybean bHLH genes including full names and predicted family membership. Additional File 5: An Excel spreadsheet showing the presumptive rice and Arabidopsis orthologs of the soybean bHLH genes. Additional File 6: An Excel spreadsheet containing a list of bHLHs enriched for expression in seed or nodules. Additional File 7: The alignment of 319 soybean bHLH genes in PHYLIP format for computer analysis. Additional File 8: A LOGO illustration of the putative Motif 40. [file 603182.f1.zip › Supplementary Material/Additional File 3.pdf]
