# Supplementary material for: A Classification of Basic Helix-Loop-Helix Transcription Factors of Soybean
Source: Int J Genomics. 2015 Feb 11;2015:603182. doi: 10.1155/2015/603182 (PMC4339708; doi:10.1155/2015/603182)
Supplement: Supplementary file 1 — As Supplementary Material we have included the following. Addititional File 1: an Excel spreadsheet file that includes names and expression data for all of the soybean bHLH-encoding gene models. Additional File 2: A list of soybean gene models excluded from the analysis due to an incomplete bHLH domain or predicted in-frame stop codon. Additional File 3: A full-text alignment of all 319 soybean bHLH domains in pdf format. Additional File 4: a detailed phylogenetic tree of the soybean bHLH genes including full names and predicted family membership. Additional File 5: An Excel spreadsheet showing the presumptive rice and Arabidopsis orthologs of the soybean bHLH genes. Additional File 6: An Excel spreadsheet containing a list of bHLHs enriched for expression in seed or nodules. Additional File 7: The alignment of 319 soybean bHLH genes in PHYLIP format for computer analysis. Additional File 8: A LOGO illustration of the putative Motif 40. [file 603182.f1.zip › Supplementary Material/Additional File 8.pdf]

Additional File 8. New bHLH motif (Motif 40).

A: Sequence logo representation of consensus motif

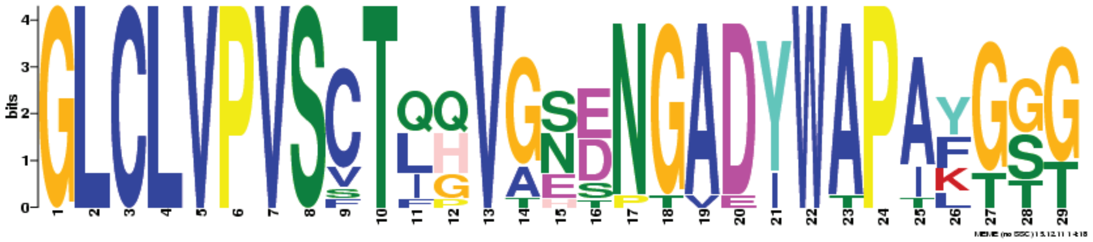

B. MEME alignment of sequences used to discover motif

Name	Start	*p*-value	Sites
Glyma08g21770.2:(Glyma08g21770.2|PACid:16272011	287	5.24e-38	KDKPSDLKSR GLCLVPVSCTQHVGNENGADYWAPAYGSG F
Glyma08g21770.1:(Glyma08g21770.1|PACid:16272010	289	5.24e-38	KDKPSDLKSR GLCLVPVSCTQHVGNENGADYWAPAYGSG F
Glyma07g02120.3:(Glyma07g02120.3|PACid:16265869	289	5.24e-38	KDKPRDLKSR GLCLVPVSCTQHVGNENGADYWAPAYGSG F
Glyma07g02120.2:(Glyma07g02120.2|PACid:16265868	287	5.24e-38	KDKPRDLKSR GLCLVPVSCTQHVGNENGADYWAPAYGSG F
Glyma07g02120.1:(Glyma07g02120.1|PACid:16265867	289	5.24e-38	KDKPRDLKSR GLCLVPVSCTQHVGNENGADYWAPAYGSG F
Glyma08g04110.3:(Glyma08g04110.3|PACid:16269892	316	2.62e-36	EEANKDLRSR GLCLVPVSCTLQVGSDNGADYWAPAFGGG FR
Glyma08g04110.1:(Glyma08g04110.1|PACid:16269890	343	2.62e-36	EEANKDLRSR GLCLVPVSCTLQVGSDNGADYWAPAFGGG FR
Glyma05g35610.1:(Glyma05g35610.1|PACid:16260811	325	2.62e-36	EEPKKDLRSR GLCLVPVSCTLQVGSDNGADYWAPAFGGG FR
Glyma07g09180.3:(Glyma07g09180.3|PACid:16266721	220	2.57e-35	EEPKKGLRSR GLCLVPVSCTLQVGSDNGADYWAPALGGG FR
Glyma07g09180.1:(Glyma07g09180.1|PACid:16266719	303	2.57e-35	EEPKKGLRSR GLCLVPVSCTLQVGSDNGADYWAPALGGG FR
Glyma02g15520.3:(Glyma02g15520.3|PACid:16248175	124	5.33e-29	GVEPCSLRSR GLCLVPVSVTIGVAESNGADIWAPIKTTT SPKFEKDVSQ
Glyma02g15520.1:(Glyma02g15520.1|PACid:16248173	126	5.33e-29	GVEPCSLRSR GLCLVPVSVTIGVAESNGADIWAPIKTTT SPKFEKDVSQ
Glyma07g32980.1:(Glyma07g32980.1|PACid:16268553	126	5.83e-29	GMEPCSLRSR GLCLVPVSFTIGVAETNGADIWAPIKTTT SPKFEKDVSQ
Glyma06g40760.1:(Glyma06g40760.1|PACid:16264777	458	6.22e-24	EGPKQDLRSR GLCLVPVSSTFPVTHEPTVEYWTPTFGGT YR
